# STIL: a multi-function protein required for dopaminergic neural proliferation, protection, and regeneration

**DOI:** 10.1038/s41420-019-0172-8

**Published:** 2019-04-25

**Authors:** Lei Li, Congcong Liu, Aprell L. Carr

**Affiliations:** 1 Department of Biological Sciences, University of Notre Dame, Notre Dame, IN 46556 USA; 2 Center for Reproductive Medicine, Shandong University, Jinan, 250001 China

**Keywords:** Cell death in the nervous system, Mechanisms of disease

## Abstract

Degeneration of dopaminergic (DA) neurons in the brain is the major cause for Parkinson’s disease (PD). While genetic loci and cellular pathways involved in DA neuron proliferation have been well documented, the genetic and molecular and cellular basis of DA cell survival remains to be elucidated. Recently, studies aimed to uncover the mechanisms of DA neural protection and regeneration have been reported. One of the most recent discoveries, i.e., multi-function of human oncogene SCL/TAL interrupting locus (*Stil*) in DA cell proliferation, neural protection, and regeneration, created a new field for studying DA cells and possible treatment of PD. In DA neurons, *Stil* functions through the *Sonic hedgehog* (*Shh*) pathway by releasing the inhibition of SUFU to GLI1, and thereby enhances *Shh*-target gene transcription required for neural proliferation, protection, and regeneration. In this review article, we will highlight some of the new findings from researches relate to *Stil* in DA cells using zebrafish models and cultured mammalian PC12 cells. The findings may provide the proof-of-concept for the development of *Stil* as a tool for diagnosis and/or treatment of human diseases, particularly those caused by DA neural degeneration.

## FACTS

- *Stil* is a human oncogene which may also function as ON-OFF switches that regulate the death and survival of neurons
- *Stil* is required for neural proliferation and regeneration
- *Stil*-mediated *Shh* signaling transduction protects dopaminergic neurons in response to toxic drug treatment

## OPEN QUESTIONS

- How to control the expression of *Stil* mRNA in neurons?
- Does *Stil* play a role in regulation of neural function?
- Can *Stil* be developed as a molecular marker for diagnosis of neural degeneration?

## Introduction

The SCL/TAL interrupting locus (*Stil*) is a human oncogene that was originally identified from human T-cell lymphoblastic leukemia, where it functions as a hematopoietic transcription factor^1^. The sequence of *Stil* gene is highly conserved in vertebrate species^2^. In humans, the *Stil* transcript is comprised of 18 exons which yield a peptide of 1287 amino acids in 143-KD^2,3^. While STIL does not share any known structural motifs with other proteins, analyses of the human STIL sequence revealed some similarities between STIL and the C-terminus of cytokine TGF-β^4^ and the C-terminus of centriole duplication factor Ana2^5^. STIL is expressed in all cell types examined so far^6–10^. In prometaphase-synchronized cells and mouse tumor cell lines, STIL functions as a cell cycle checkpoint protein that regulates the transition of mitotic entry during cell proliferation^11^. In developing human cancer cells, STIL is localized to the pericentriolar region of the centrosomes, where it regulates in spindle pole positioning as well as centriole formation and duplication^12–14^.

**Fig. 1:**
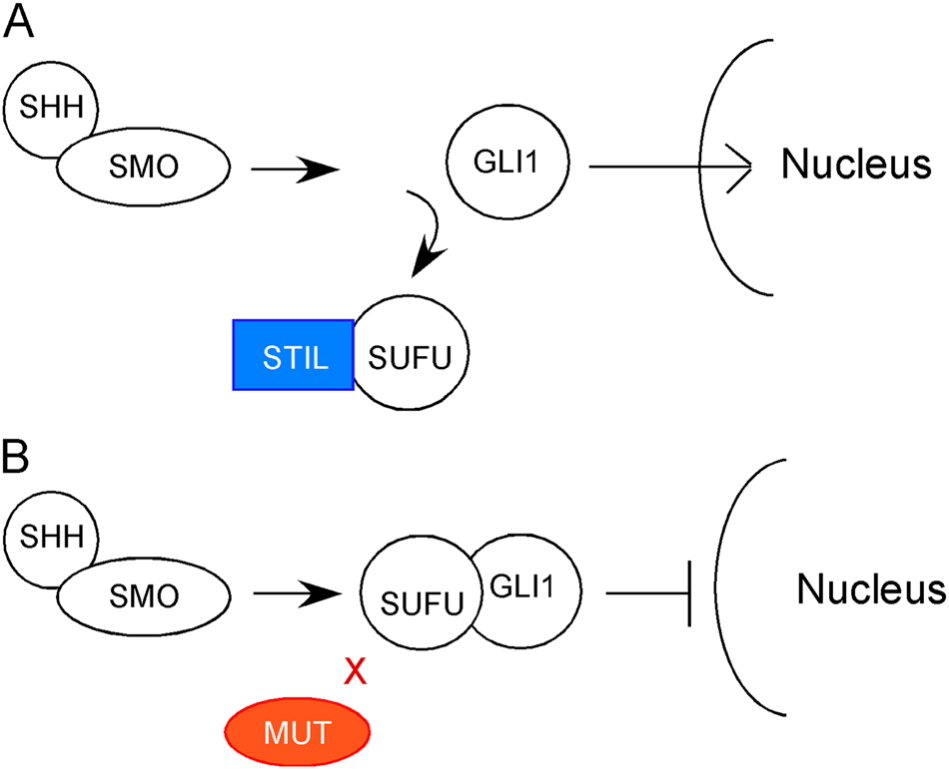
Diagrams that illustrate the roles of STIL in the *Shh* pathway. **a** Normally, STIL binds SUFU, releases SUFU’s suppression on GLI1, then GLI1 can be translocated to the nucleus for gene transcription. **b** When STIL is mutated, *Shh* down-stream signaling transduction cannot be completed because the SUFU-GLI1 heterodimers cannot enter the nucleus for gene transcription. (Modified from reference^15^)

While ample proof of STIL functions in cancer can be found in the literature, evidence also exists that STIL is involved in the development and function of the nervous system. In mouse, for example, functional expression of *Stil* is required for setting the midline and left-right polarity of the central nervous system (CNS)^15^. In mutants which lack proper *Stil* expression and/or with defective structure of *Shh* receptors, the embryos develop abnormally and die at age of E11. Using molecular genetic approaches, Izraeli et al.^15^ and Kasai et al.^16^ demonstrated that *Stil* functions in the *Sonic hedgehog* (*Shh*) pathway. Specifically, *Stil* regulates the transcription of *Shh*-target gene *Gli1*. Normally, GLI1 forms heterodimers after binding to cytoplasmic protein SUFU. The heterodimers cannot be translocated to the nucleus thereby the transcription of *Gli1* gene is inhibited. When STIL is expressed, STIL binds SUFU, frees GLI1 from SUFU repression. Then, GLI1 can enter the nucleus, and gene transcription can start. If *Stil* is mutated, the transcription of *Gli1* cannot be initiated (Fig. 1).

Recent studies have shown that *Stil* plays important role for proliferation, survival, regeneration, and possibly functions in dopaminergic (DA) neurons^17,18^. In humans, DA-mediated neural transduction is required for integration of sensory-motor signals and the control of movement^19–22^. The lack of DA cells and/or malfunction of DA neural networks may lead to a variety of neurological diseases, such as PD^23–26^. In this article, we will review some of the findings relate to the roles of *Stil* in DA cells. The data presented in this article are obtained from studies conducted in zebrafish models and cultured mammalian DA cell lines. The results may provide insight for future development of *Stil* as a potential bio-marker for better understanding the molecular and cellular mechanisms underlying human neurological disorders.

## Multi-function of STIL in DA cells

### STIL protects DA cells

While most the studies related to *Stil* were carried out using cultured cancer cell lines and mouse models^9,11,15,16,27–29^, research that characterize *Stil* mutations that cause dramatic phenotypes (e.g., embryonic lethality, nervous system malfunction) can also be found by using other species, such as zebrafish. Pfaff et al.^30^ reported that in zebrafish, STIL is highly expressed in proliferating cells. During metaphase, STIL is concentrated in areas near the poles of the mitotic spindles. In *cassiopeia* (designated *csp*, which is a homolog of human *Stil*) mutants, the *Stil* gene is mutated, and the organization of spindle pole in proliferating cells is completely disrupted. Often, the mutants lack one or both centrosomes. As a consequence, the embryos develop abnormally and die between 7 and 10 days post-fertilization. In another study, Li and Dowling^31^ demonstrated that in zebrafish *night blindness b* (designated *nbb*; also a homolog of human *Stil*) mutants, not only the development but also the function of the CNS are interrupted. In homozygous *nbb* mutants, massive cell degeneration occurs in the developing brain, and the embryos die before 7 days post-fertilization. In heterozygous *nbb* mutants, animals are viable but after 9 months of age, abnormalities are found in the CNS, e.g., the number of retinal DA cells is decreases, and the animals become night blind particularly after prolonged dark adaptation. In addition, in *nbb* mutants the susceptibility of DA cells to neurotoxin is increased^17^, and the rate of DA cell regeneration is decreased^32^. Molecular cloning and sequence analysis of the *nbb* locus revealed a nucleotide substitution at a donor-splicing site in the *Stil* transcript, which results in a predicted truncated peptide with 301 amino acids^17^.

**Fig. 2:**
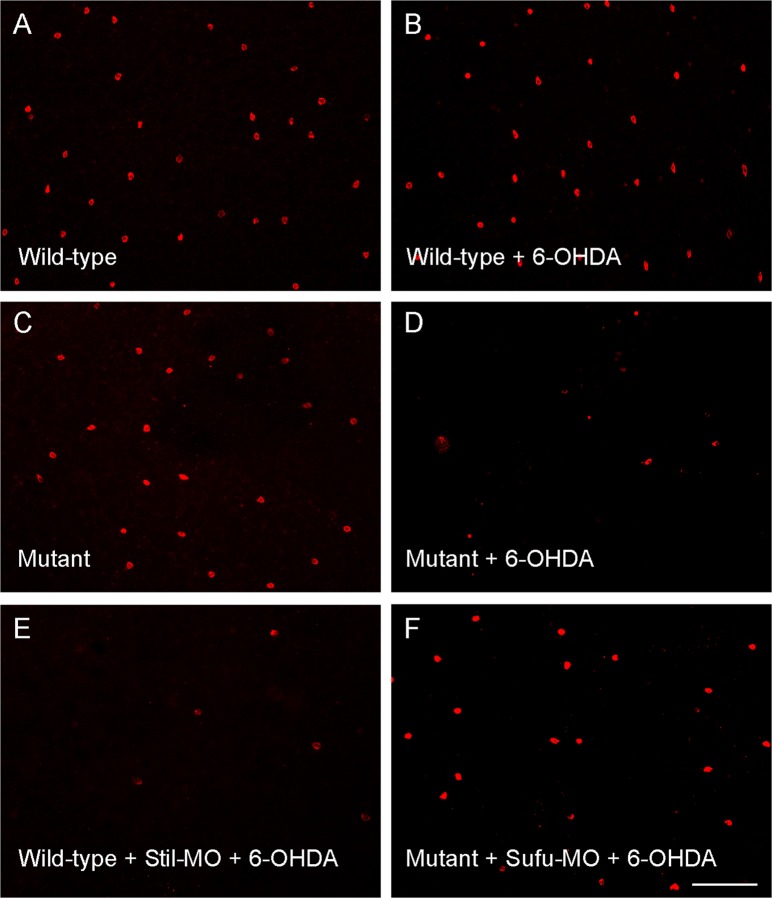
Fluorescent images of flat-mounted zebrafish retinas that show the DA cells (labeled with antibodies against tyrosine hydroxylase) after treatment with sub-toxic 6-OHDA. **a**, **b** Wild-type retinas that received sham or sub-toxic 6-OHDA treatment. No differences in the number of DA cells were observed. **c**, **d** Mutant retinas that received sham or sub-toxic 6-OHDA treatment. The number of DA cells was decreased after drug treatment. **e**, **f** Wild-type and mutant retinas that received sub-toxic 6-OHDA injections, but previously treated with *Stil*- and *Sufu*-specific MOs, respectively. Note the increase of drug susceptibility (decreases in cell survival) in wild-type fish and the decrease of drug susceptibility (increases in cell survival) in mutant fish. Scale bar, 100 μm. (Modified from reference^17^)

To investigate if the expression of *Stil* is required for maintaining the DA cells (e.g., whether or not the decrease of DA cells in *nbb* mutants is due to age-related neural degeneration), Li et al.^17^ examined the survival of DA neurons in zebrafish retinas in response to treatment with DA-specific neurotoxins, such as 6-hydroxydopamine (6-OHDA)^33,34^. They found that deficiency in *Stil* expression led to increases in toxic susceptibility in DA neurons. In wild-type zebrafish, for example, treatment with sub-toxic levels of 6-OHDA (0.33 µg/μl) produced no obvious effects on the survival of DA cells (Fig. 2a, b). In *nbb* mutants, however, the same treatment led to significant DA degeneration (Fig. 2c, d). The susceptibility of DA cells to neural toxins can be reversed by manipulating the levels of *Shh* signaling transduction, e.g., by inhibition of STIL expression using *Stil*-specific morpholinos (MOs), or increase of *Shh* signaling transduction by knocking-down SUFU expression with *Sufu*-specific MOs. In zebrafish, treatment with gene-specific MOs can efficiently knock-down their protein expressions^35,36^. In wild-type zebrafish, decreases in STIL expression (by *Stil*-MO) led to increases of the susceptibility of DA cell to sub-toxic levels of 6-OHDA. As a consequence, DA cell degeneration was observed when treated with sub-toxic levels of 6-OHDA (Fig. 2e). In contrast, in *nbb* mutants, inhibition of SUFU expression (by *Sufu*-MO, which then lifted its suppression to GLI1, and increased *Gl1* transcription) lowered the susceptibility of DA cells to neural toxins and protected the DA cells after drug treatment (Fig. 2f).

### STIL promotes DA regeneration

**Fig. 3:**
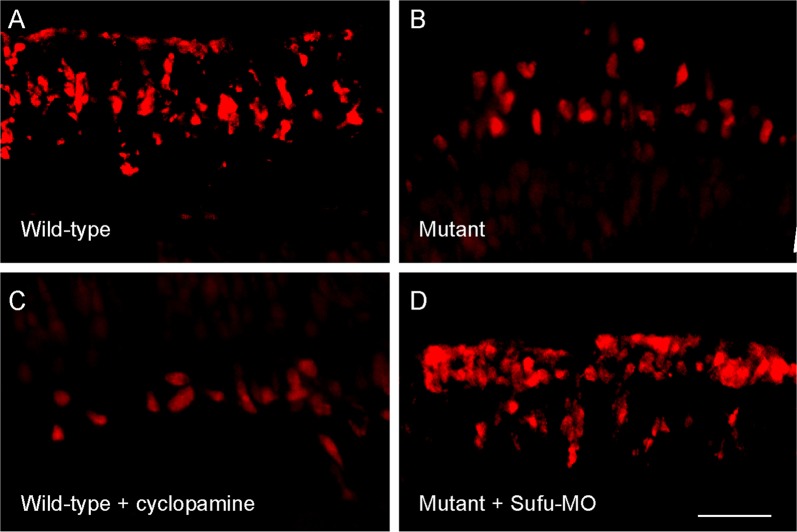
Fluorescent images of retinal sections that show DA cell regeneration. **a**, **b** Sections from wild-type and *nbb* retinas. Note the decrease of PCNA labeling in the mutant. **c** A section from cyclopamine-treated wild-type fish. Note the decrease in the number of PCNA-positive cells. **d** A section from *nbb* fish that received *Sufu*-MO injection. Note the increase in the number of PCNA-positive cells. Scale bar, 100 μm. (Modified from reference^32^)

Zebrafish are capable to replace lost neurons by reactivation of stem cell-like glia cells^37–42^. In a research aimed to distinguish whether or not the decrease of DA neurons in *nbb* mutants is related to DA neural regeneration, Sun et al. (2014a) examined the proliferation of DA cells in adult zebrafish retinas after 6-OHDA induced neural degeneration. In this study, toxic levels of 6-OHDA (5.0 µg/μl) was applied in order to effectively induce the degeneration of DA cells. After a few days of 6-OHDA treatment, the samples were collected and labeled with antibodies against proliferating cell nuclear antigen (PCNA), a marker protein that only expresses in proliferating cells^38,43^. In wild-type animals, after drug-induced cell death of DA cells, robust regeneration of DA cells occurred, e.g., PCNA-positive cells could be readily identified (Fig. 3a). In *nbb* retinas, however, in response to the same treatment, the rate of DA cell regeneration was decreased, e.g., the number of PCNA-positive cells was only 30–40% of the number of PCNA-positive cells counted in control retinas (Fig. 3b).

Because *Stil* functions in the *Shh* pathway, Sun et al.^32^ tested if the survival of DA cells is affected by *Shh* signaling transduction. The experiments were performed in wild-type animals in which the transduction of *Shh* signaling was blocked by steroidal alkaloid cyclopamine, and in *nbb* mutants in which the transduction of *Shh* signaling was enhanced by knock-down of SUFU expression with *Sufu*-MO. Previous studies have demonstrated that cyclopamine, an antagonist of SMO receptor in the *Shh* pathway, can efficiently block the *Shh* signaling transduction and lower the transcription of *Gli1*^44,45^. In wild-type fish, treatment with cyclopamine decreased the rate of DA cell regeneration after 6-OHDA induced degeneration (Fig. 3c). In *Sufu*-MO treated *nbb* fish, after 6-OHDA treatment the number of cells that express PCNA-positive cells was increased to levels similar as seen in wild-type animals. Together, the data suggest that increases in *Shh* signaling transduction may rescue the defects in DA cell regeneration due to the expression of *nbb* mutation (Fig. 3d).

### STIL induces mammalian DA cell proliferation

The roles of STIL expression in mammalian DA cell proliferation were evaluated using cultured PC12 cells^18^. The PC12 cells were originally derived from rat adrenal pheochromocytoma cells^46–48^. Under normal culture conditions and with inductions by nerve growth factor (NGF), PC12 precursor cells can be proliferated and differentiated into DA cells^49,50^. The overexpression of *Stil* (by transfection with full-length human *Stil* sequence) increased the rate of cell proliferation of PC12 cells, by approximately 30% compared to control cells. In contrast, knock-down of *Stil* function (by *Stil*-specific shRNA) reduced the rate of PC12 cell proliferation, to a level approximately 45% of controls^18^.

To investigate whether or not STIL also functions in the *Shh* pathway as a de-repressor for proper signaling transduction in PC12 cells, Sun et al.^51^ examined the co-expression of STIL and *Shh* down-stream proteins, such as SUFU and GLI1, in PC12 cells. They found that both SUFU and GLI1 could be detected by anti-STIL antibody pull-down experiments. STIL and SUFU could also be detected by using anti-GLI1 antibody pull-down assays. These results provide evidence for the conserved roles of STIL in the *Shh* pathway in mammalian DA cells. The roles of *Stil* in *Shh* pathway in PC12 cells can also be demonstrated using pharmacological approaches. Carr et al.^18^ proposed that down-regulation of *Shh* signaling transduction by cyclopamine would mimic the proliferation defects caused by *Stil*-shRNA knock-down. They found that treatment with cyclopamine indeed reduced the proliferation rate of PC12 cells. Interestingly, overexpression of *Stil* was able to maintain a significant higher growth rate in the presence of cyclopamine. This suggests that when the initial *Shh* signaling is blocked, some of the down-stream signaling transduction could be continued by exogenous expression of STIL. That is, STIL binds SUFU and releases GLI1, then GLI1 enters the nucleus and starts gene transcription.

By the same token, whether or not the up-regulation of *Shh* signaling has a role on PC12 cell proliferation depends on the availability of STIL. This can be tested using pharmacologic approaches with purmorphamine while the expression of *Stil* is down- or up-regulated. It is known that purmorphamine functions as a SMO receptor agonist in the *Shh* pathway^52–54^. The application of purmorphamine increased the rate of PC12 cell proliferation^18^. However, when the expression of *Stil* mRNA was blocked by *Stil*-shRNA, purmorphamine could not fully rescue the defects in PC12 cell proliferation^18^. That suggests that activation the up-stream *Shh* signaling transduction is not able to bring the proliferation rate to control levels if the expression of down-stream genes (e.g., *Stil*) is inhibited. Based on these observations, Carr et al.^18^ concluded that STIL may induce cell proliferation by propagating the intracellular *Shh* signal for subsequent transcription of *Shh*-target genes, which are cell cycle specific.

### STIL facilitates mammalian DA neural growth

Carr et al.^18^ examined the distribution of STIL in PC12 cells. They found that STIL was expressed in both developing and NGF-induced mature cells. In differentiated mature cells, STIL was ubiquitous expressed in the cytoplasm, nucleus, as well as in the neurites. The result is not unexpected, considering the primary roles of *Stil* in mitotic entry, spindle pole organization, centriole formation and duplication. The expression of STIL in mature cells indicates novel non-proliferative functions of STIL in post-mitotic and differentiate cells, such as neural survival, neurite extension, synthesis or release of neurotransmitters. This supports the notion that *Stil* is involved in the regulation of microtubule elongation^55^.

While STIL is required for DA cell prolifaration and growth, the expression of *Stil* is not involved in PC12 cell neural differentiation. After NGF induction, out-growth of neurites was observed in PC12 cells regardless the levels of *Stil* expression. Also, the rate neurite growth was indistinguishable among the cells in which the expression of *Stil* was knocked-down (by transfection with *Stil*-shRNA) or the expression of *Stil* mRNA was up-regulated (by transfection with plasmids that contained the full-length of human *Stil* DNA)^18^.

### STIL in regulation of DA cell drug susceptibility

**Fig. 4:**
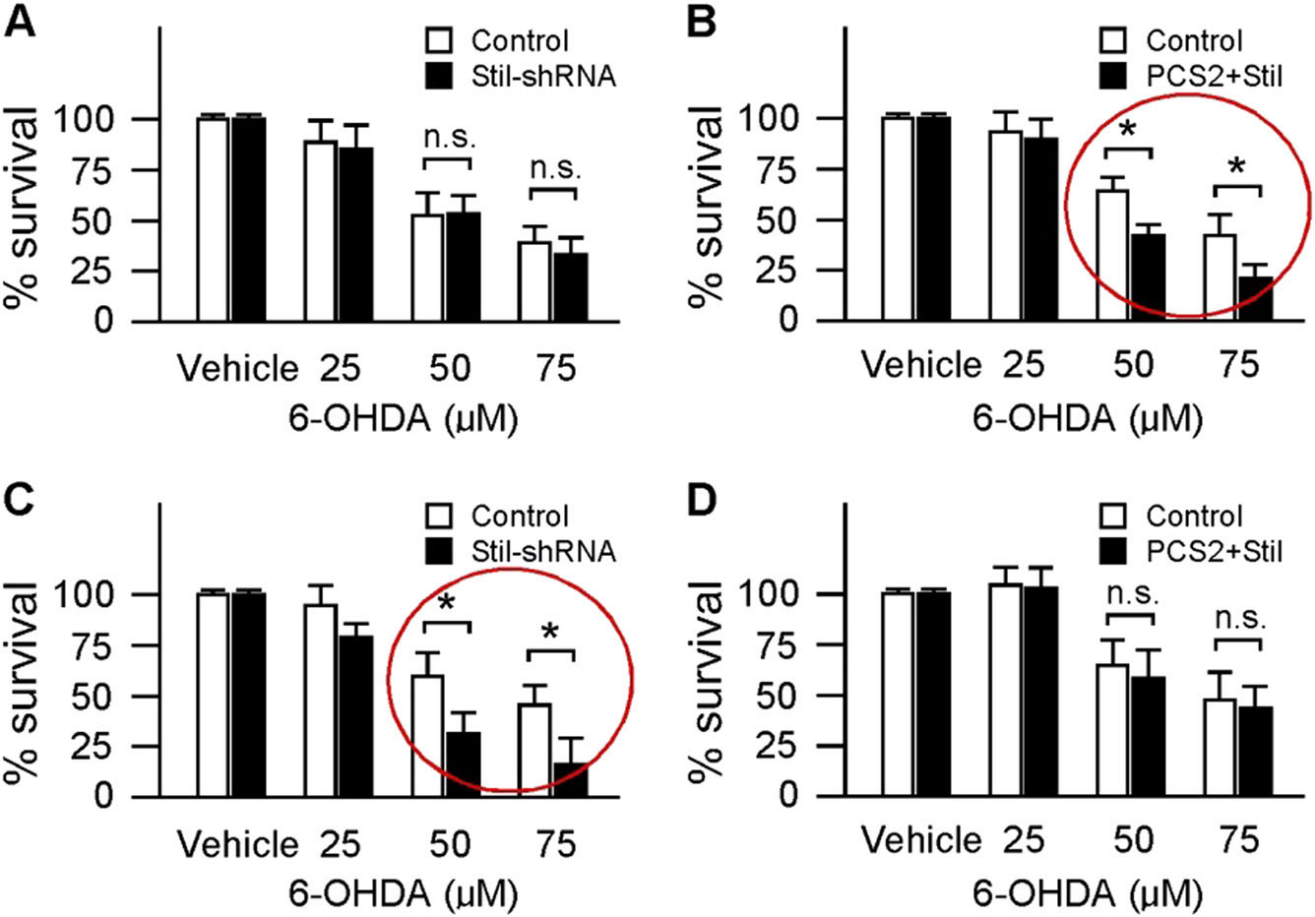
Effects of *Stil* expression on PC12 cell drug susceptibility. The rate of survival under control conditions (treated with vehicle solution) is normalized to 100%. **a**, **b** Percentage of cell survival after 6-OHDA treatment in proliferating cells. Note the increase of drug susceptibility after the overexpression of *Stil* (red circle). **c**, **d** Percentage of cell survival after 6-OHDA treatment in mature cells. Note the increase of drug susceptibility when the expression *Stil* is down regulated (red circle). Asterisks indicate statistical differences, *p* < 0.05; *ns*, not significant (Modified from reference^56^)

The effects of *Stil* expression on neural protection have been evaluated in cultured PC12 cells^56^. It appears that, to some extent, STIL may function as a “two-edged blade” that involves in both neural proliferation and degeneration, depends on the status of the cell. In proliferating PC12 cells, knock-down of STIL expression (by shRNA) produced no obvious effect on cells’ drug toxicity, e.g., cell death occured when treated with high doses of 6-OHDA, similar as observed in control cells (Fig. 4a). However, overexpression of *Stil* (by transfection of full-length human *Stil* DNA) increased the susceptibility of PC12 cells to neurotoxins, and when exposed to higher doses of 6-OHDA (e.g., 50 µM, 75 µM), excessive cell death was observed (Fig. 4b). In differentiated PC12 cells, *Stil* functions in opposite ways. That is, overexpression of *Stil* produced no effect on cell’s drug susceptibility as compared to control cells, but knock-down of *Stil* increased the drug susceptibility and when treated with higher doses of 6-OHDA, the rate of cell death was increased as compared to control cells (Fig. 4c, d).

## Conclusion

In summary, this paper provides evidence that through the *Shh* signaling transduction pathway the human oncogene *Stil* plays novel roles in DA cells. The expression of *Stil* is required for neural proliferation, survival, and regeneration. Considering the conserved roles of *Stil* in cell proliferation, the involvement of *Stil* in the *Shh* pathway, and the effect of *Shh* signaling in drug resistance, it is conceivable that *Stil* may function as an ON-OFF switch that controls the outcome of *Shh* signaling transduction, which in turns, regulates the fate of individual cell groups, for example, to be developed or undergo apoptosis. If this model proves true, then *Stil* may be considered as a bio-marker for basic and translational researches relate to human health, i.e., by analysis of the expression of *Stil* mRNA, the fate of certain cell types (e.g., cancer cells or neurons) can be predicted (e.g., proliferation, survival, or apoptosis). Future researches on *Stil* shall include but not limit to the characterization of the regulation of *Stil* expression in cancer cells and neurons, the interplay between *Stil* products and hormones that regulate cancer growth and neural degeneration, and the mechanisms of *Stil*-mediated *Shh* signaling transduction in drug resistance. At some point, one shall be able to control the molecular ON-OFF switch for *Stil* expression, and this will lead to either cell proliferation (e.g., neural regeneration) or apoptosis (e.g., cancer cells). It is expected that in the future *Stil* may be developed as a tool for diagnosis and/or treatment of human diseases.
